# Presence of a Prophage Determines Temperature-Dependent Capsule Production in *Streptococcus pyogenes*

**DOI:** 10.3390/genes7100074

**Published:** 2016-09-24

**Authors:** Leslie Brown, Jeong-Ho Kim, Kyu Hong Cho

**Affiliations:** 1 Department of Biology, Indiana State University, Terre Haute, IN 47809, USA; lesliejobrown@gmail.com; 2 Department of Biochemistry and Molecular Medicine, The George Washington University School of Medicine and Health Science, Washington, DC 20037, USA; jh_kim@email.gwu.edu

**Keywords:** *Streptococcus pyogenes*, Hyaluronic acid capsule, Capsule thermoregulation, CovRS, Prophages

## Abstract

A hyaluronic acid capsule is a major virulence factor in the pathogenesis of *Streptococcus pyogenes*. It acts as an anti-phagocytic agent and adhesin to keratinocytes. The expression of the capsule is primarily regulated at the transcriptional level by the two-component regulatory system CovRS, in which CovR acts as a transcriptional repressor. The *covRS* genes are frequently mutated in many invasive strains, and a subset of the invasive CovRS mutants does not produce a detectable level of the capsule at 37 °C, but produces a significant amount of the capsule at sub-body temperatures. Here, we report that a prophage has a crucial role in this capsule thermoregulation. Passaging CovR-null strains showing capsule thermoregulation using a lab medium produced spontaneous mutants producing a significant amount of the capsule regardless of incubation temperature and this phenotypic change was caused by curing of a particular prophage. The lab strain HSC5 contains three prophages on the chromosome, and only ΦHSC5.3 was cured in all spontaneous mutants. This result indicates that the prophage ΦHSC5.3 plays a crucial role in capsule thermoregulation, most likely by repressing capsule production at 37 °C.

## 1. Introduction

*Streptococcus pyogenes* causes diverse human diseases ranging from mild, self-limiting superficial infections, such as strep throat and impetigo, to toxigenic or invasive diseases, such as streptococcal toxic shock syndrome and necrotizing fasciitis. A variety of *S. pyogenes* virulence factors is involved in causing these diverse outcomes. A major virulence factor in the pathogenesis of *S. pyogenes* is the capsule that is composed of hyaluronic acid, the same component in human connective tissues. The capsule contributes to the pathogenesis of *S. pyogenes* as an antiphagocytic factor [1,2,3] and adhesin to keratinocytes [3,4,5,6].

Like other virulence factors, capsule production in *S. pyogenes* is tightly regulated. The capsule is produced by the products of three genes in the capsule operon, *hasA*, *hasB*, and *hasC*, encoding hyaluronan synthase, UDP-glucose 6-dehydrogenase, and UDP-glucose pyrophosphorylase, respectively. These genes are transcribed as a single transcript by a promoter in front of the *hasA* gene. The transcription of the capsule operon is regulated by the two-component regulatory system CovRS (also known as CsrRS) [7]. Upon activation, the sensor kinase CovS autophosphorylates, then transfers the phosphate to the response regulator CovR, which acts as a transcriptional repressor. When phosphorylated, CovR binds in the promoter region of the capsule operon and represses transcription of the capsule genes [8]. CovR also represses the transcription of other virulence factors such as streptokinase, streptolysin S and SpeMF, a mitogenic factor [9].

We previously reported that most invasive clinical isolates of *S. pyogenes* produce the capsule but none of the strains causing superficial infections produce it [10]. Among the capsule producers, capsule production of some clinical isolates is regulated by environmental temperature [10]. These strains produce a large amount of capsules at sub-body temperature, but no detectable level at 37 °C. Capsule thermoregulation occurs at a post-transcriptional level, so the repression of capsule transcription by CovR should be released prior to capsule thermoregulation. As expected, all of the invasive strains showing capsule thermoregulation have a mutation in the *covR* or *covS* gene [10]. Since the capsule is an antiphagocytic factor that helps invasive infections, the phenotype of capsule thermoregulation only producing a large amount of the capsule at sub-body temperature is puzzling. However, this study shows a possibility that the strains showing capsule thermoregulation can be converted to the ones producing the capsule regardless of environmental temperature.

Another streptococcal factor involved in capsule thermoregulation is CvfA [10]. CvfA null mutants lose capsule thermoregulation, and produce a lot of capsules regardless of environmental temperature. CvfA is an endoribonuclease, which is also known as RNase Y, but it is not directly involved in the degradation of the capsule transcript because the amounts of capsule transcript between the wild-type and the CvfA null mutants at 37 °C are not different, even though the amounts of capsule production are different. This discrepancy might indicate that there is another factor in capsule thermoregulation whose transcript level is influenced by CvfA. Previously, we noticed that spontaneous capsule overproducers arose during handling CovR mutants. Thus, we screened for spontaneous capsule overproducing mutants at 37 °C by passaging a *covR* mutant in a laboratory medium, sequenced the whole genomes of the mutants to find mutation sites, and discovered that a prophage plays a crucial role in capsule thermoregulation.

## 2. Materials and Methods

### 2.1. Bacterial Strains and Growth Conditions

*S. pyogenes* HSC5 was used for the experiments and strain construction in this study [11]. Todd Hewitt medium (BBL) with 0.2% yeast extract (THY media) was used to cultivate *S. pyogenes*. For growth in liquid media, *S. pyogenes* was cultured at 37 °C in sealed tubes without shaking. To produce solid media, Bacto agar was added to a final concentration of 1.4% (wt/vol). *S. pyogenes* on agar plates (solid media) was incubated in anaerobic condition using the Gas Pak EZ Anaerobe containment system (BD, Franklin Lakes, NJ, USA). *Escherichia coli* Top10 (Invitrogen) was used for plasmid construction. *E. coli* was cultured in Luria-Bertani broth (LB) at 37 °C with shaking. When appropriate, antibiotics were added to the media at the following concentrations unless specified; kanamycin 50 μg/mL for *E. coli* and 500 μg/mL for *S. pyogenes*. Strains used in this study are listed in Table 1.

### 2.2. Quantitation of *S. pyogenes* Capsule

The capsule amount produced by an *S. pyogenes* colony was measured using a spectrophotometric assay as described previously [10]. Briefly, an overnight culture of *S. pyogenes* grown in THY medium was diluted with phosphate-buffered saline (PBS) and plated on THY agar plates to have less than 30 colonies. These plates were incubated anaerobically at 37 °C overnight to grow colonies and then incubated further at 25 °C or 37 °C for six to eight hrs. Afterward, a single colony was suspended in 1 mL of sterile deionized water and capsules were dissociated from the cells through vigorous agitation by the FastPrep 24 (MP Biomedicals, Santa Ana, CA, USA) without using glass beads at a speed of 6.0 m/s for 20 s. A portion of the solution was taken to determine the number of colony-forming units (CFUs) per mL by plating after serial dilution with the PBS buffer. Cells were removed from the remaining solution by centrifugation. The capsule-containing supernatant was mixed with the equal volume of capsule-staining reagent (20 mg of the cationic carbocyanine dye, 1-ethyl-2-[3-(1-ethylnaphtho-[1,2-d]thiazolin-2-ylidene)-2-methylpropenyl]naptho-[1,2-d]thiazolium bromide (Sigma Chemical Co., St. Louis, MO, USA) and 60 µL of glacial acetic acid in 50 mL of 50% formamide. The quantity of the capsule was calculated by measuring the absorbance of the mixture at an OD_640_ and comparing with a standard curve generated with known concentrations of hyaluronic acid. The amount of capsules per colony-forming unit (femtogram per CFU) was then calculated using the capsule quantity and CFU count. Capsule quantitation was performed at least in triplicate for each sample, and at least twice per each strain.

### 2.3. Targeted Insertional Disruption of the *L897_07695* Gene

The *L897_07695* gene was disrupted by a single-crossover homologous insertion as follows: an internal region of a gene was amplified by polymerase chain reaction (PCR) using primers AAACTGCAGTTTCCAAAATAATTGGATGCG and AAACTGCAGTGGTTCTTTGTAGTCTGGATTCG, and inserted into the suicide vector pCIV2, pUC18-based streptococcal integration vector containing the kanamycin resistance gene *aphA3* [13]. The resultant plasmid was purified and used to transform *S. pyogenes* by electroporation [14]. The desired mutant was screened by antibiotic resistance, and the disruption of the target gene was confirmed by PCR with primers annealing at a chromosomal locus near the amplified region and the multiple cloning site in the plasmid. 

### 2.4. Passaging Cells for Generating Spontaneous Capsule Thermoregulation Mutants

The HSC5 CovRIFD strain was passaged five times in 10 mL of THY media with 100 µL of stationary phase culture (12 h culture time, OD_600_ = 1.7), except for the first inoculum from a freezer stock (~10 µL). The passaged culture was diluted and plated on THY agar plates (less than 50 colonies per plate) to screen for mutants that produce a large amount of the capsule at 37 °C.

### 2.5. Whole Genome Sequencing

The chromosomes of two spontaneous capsule thermoregulation mutants were sequenced through Macrogen Inc. and compared to that of the wild-type HSC5. Chromosomes of the mutants were purified using GenElute™ Bacterial Genomic DNA Kit (Sigma Chemical Co., St. Louis, MO, USA), and high purity of the chromosomal DNA was confirmed by running an agarose gel and measuring the ratio of OD_260_/OD_280_ (>1.8). Shotgun fragment libraries of the chromosomes were generated (library type: TruSeq DNA PCR-Free) with the average fragment size of 470 bps. The sequencing of the libraries was performed with 100 paired-end sequencing through the Illumina HiSeq2000 system (Illumina, San Diego, CA, USA). The high throughput genome sequencing data of both mutants were deposited with the NCBI (National Center for Biotechnology Information) with the accession number SRP083972.

### 2.6. PCR to Determine the Curing of Prophages

To determine the presence of prophages in the *S. pyogenes* HSC5 genome, PCR was performed using the primers listed in Table 2. Chromosomal DNA was extracted with a GenElute™ Bacterial Genomic DNA Kit (Sigma Chemical Co., St. Louis, MO, USA) and used as PCR templates. Each primer pair amplifies a portion of the gene *L897_05150* in ΦHSC5.1 (A), *L897_05945* in ΦHSC5.2 (B), and *L897_07670* in ΦHSC5.3 (C), or HSC5 chromosomal DNA only when ΦHSC5.3 is cured (D), the primers bind to the chromosomal flanking genes of ΦHSC5.3: the forward primer to *uvrA* (*L897_07450*) and the reverse primer to the magnesium transporter gene *L897_07770*.

### 2.7. Phage Nomenclature

Current convention to designate prophage names was used, which consists of the bacterial host name followed by a serial number that reflects the prophage chromosomal location in clockwise order [15,16].

## 3. Results

### 3.1. Generation of Capsule Thermoregulation Mutants by in Vitro Passaging

**Figure 1:**
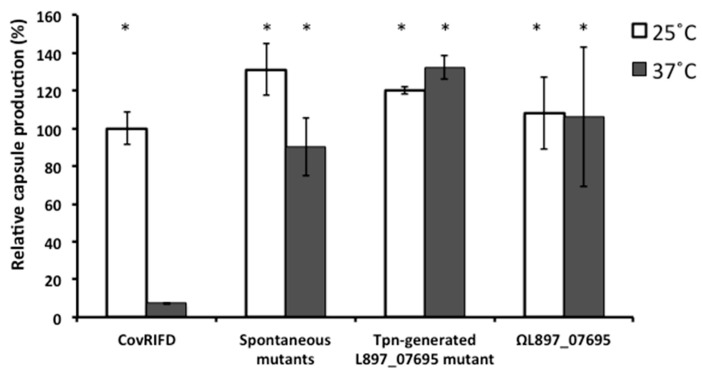
Quantitation of the capsule produced by *Streptococcus pyogenes* strains. The capsule produced by strains was quantitated and compared to that of the background strain CovRIFD incubated at 25 °C. Strains used: CovRIFD (*covR* in-frame deletion strain), the background strain showing capsule thermoregulation; spontaneous mutants, spontaneously-generated capsule thermoregulation-negative mutants; Tpn-generated L897_07695 mutant, a mutant with a transposon insertion in the *L897_07695* gene in CovRIFD; and ΩL897_07695, a strain with a targeted disruption of the *L897_07695* gene in CovRIFD. The data are the means and standard deviations of at least three independent experiments. * indicates significance (*p* < 0.01) for the difference between the amount of capsule production by strains at a given conditions and that by CovRIFD at 37 °C as calculated by the two-tailed paired Student’s *t*-test.

To study capsule thermoregulation using well-documented and genome-sequenced lab strains such as HSC5, the *covR* gene should be inactivated to confer the same phenotype as the clinically-invasive strains with capsule thermoregulation [10]. To determine if capsule thermoregulation mutants arise spontaneously, we passaged the HSC5 CovR mutant CovRIFD five times in THY media and plated on THY agar plates to screen capsule mutants. This passage generated mutants that produced a large amount of the capsule at 37 °C with the frequency of 3.8 × 10^−3^ (18 mutants out of 4729 colonies) (Figure 1).

### 3.2. A Prophage Was Cured in Spontaneous Capsule Thermoregulation-Negative Mutants

**Figure 2:**
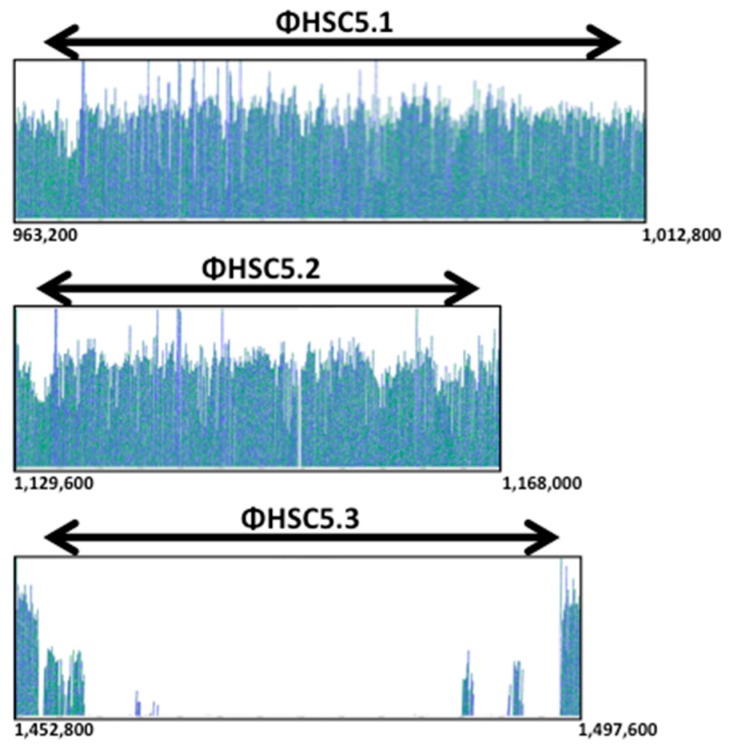
Whole genome sequencing revealed the deletion of ΦHSC5.3 in spontaneous capsule thermoregulation-negative mutants. The chromosomes of spontaneous capsule thermoregulation mutants were sequenced and compared to that of the wild-type HSC5. Each vertical line in the boxed figures is a pile-up of DNA sequences from Illumina next generation sequencing and indicates the presence of the sequence on the chromosome. The numbers under the boxed figures represent the location on the chromosome. Each horizontal arrow on top of the boxed figures indicates the position of each prophage on the chromosome: 964214–1010974 for ΦHSC5.1, 1130407–1166414 for ΦHSC5.2, and 1454753–1496038 for ΦHSC5.3. Even though ΦHSC5.3 was cured in the mutants, some sequences in ΦHSC5.3 appeared due to the existence of homologous genes in the prophages ΦHSC5.1 and ΦHSC5.2.

**Figure 3:**
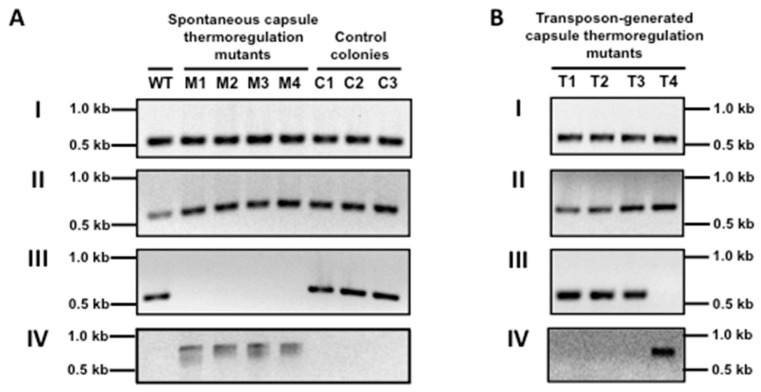
PCR assay to determine the presence of prophages on the chromosome of *Streptococcus pyogenes* strains. (**A**) Deletion of ΦHSC5.3 in spontaneous capsule thermoregulation mutants. Spontaneously generated capsule thermoregulation mutants producing a large amount of the capsule regardless of incubation temperature were screened after passaging the background strain CovRIFD in THY medium. Control colonies still displaying capsule thermoregulation on the same plates were also selected for this assay. PCR was performed to determine the presence of prophages using the primers to amplify a portion of a gene in ΦHSC5.1 (I), ΦHSC5.2 (II), or ΦHSC5.3 (III), or primers that amplify HSC5 chromosomal DNA only when ΦHSC5.3 was cured (IV). Strains used: WT (wild-type), HSC5; M1–M4 (mutant strains), randomly chosen four spontaneously arisen capsule thermoregulation-negative mutants; C1–C3 (control strains), colonies still showing capsule thermoregulation after passaging; (**B**) The existence of ΦHSC5.3 in the transposon-generated mutants. Since the excision of ΦHSC5.3 occurs spontaneously and influences capsule thermoregulation, the presence of ΦHSC5.3 in the previous transposon-generated mutants was determined through PCR. The same primers were used as those used in Figure. A. Strains used: T1–T4 (transposon-generated capsule thermoregulation mutants), a transposon insertion occurred in the gene *L897_07695* in ΦHSC5.3 (T1), at or near the ribosome binding site of *cvfA* (T2 and T3, previously described as CovRIFD:TnCvfA1 and CovRIFD:TnCvfA2, respectively [10]), or in the intergenic region (IGR) immediately upstream of the capsule operon (T4).

We chose two mutants randomly and sequenced their whole genomes with a next-generation sequencing method. The sequencing revealed several mutations in both strains, which are listed in Table S1. The mutations in both mutants were the deletion of a prophage (Figure 2) and mutations in the genes of the pore-forming toxin streptolysin O (missense mutation, L→F), *L897_04020* encoding a putative collagen-like cell surface protein (10 bps or 5 bps deletion), and *L897_07135* encoding PTS mannose transporter subunit II AB (missense mutation, V→F). Next, we examined all eighteen spontaneous mutants through PCR to detect the deletion of the prophage and through sequencing to detect the mutations in those genes. These assays resulted in all eighteen spontaneously-generated mutants having the deletion of the prophage, while control colonies producing no detectable level of capsules on the same plate still possessed the prophage (Figure 3A). However, not all of the capsule mutants had the mutations in the genes of *slo*, *L897_04020*, and *L897_07135*. Taken together, these results indicate that the deletion of the prophage in the capsule thermoregulation mutants is linked to their phenotypic change of capsule production at 37 °C.

### 3.3. The Prophage ΦHSC5.3 Influences Capsule Thermoregulation

**Figure 4:**
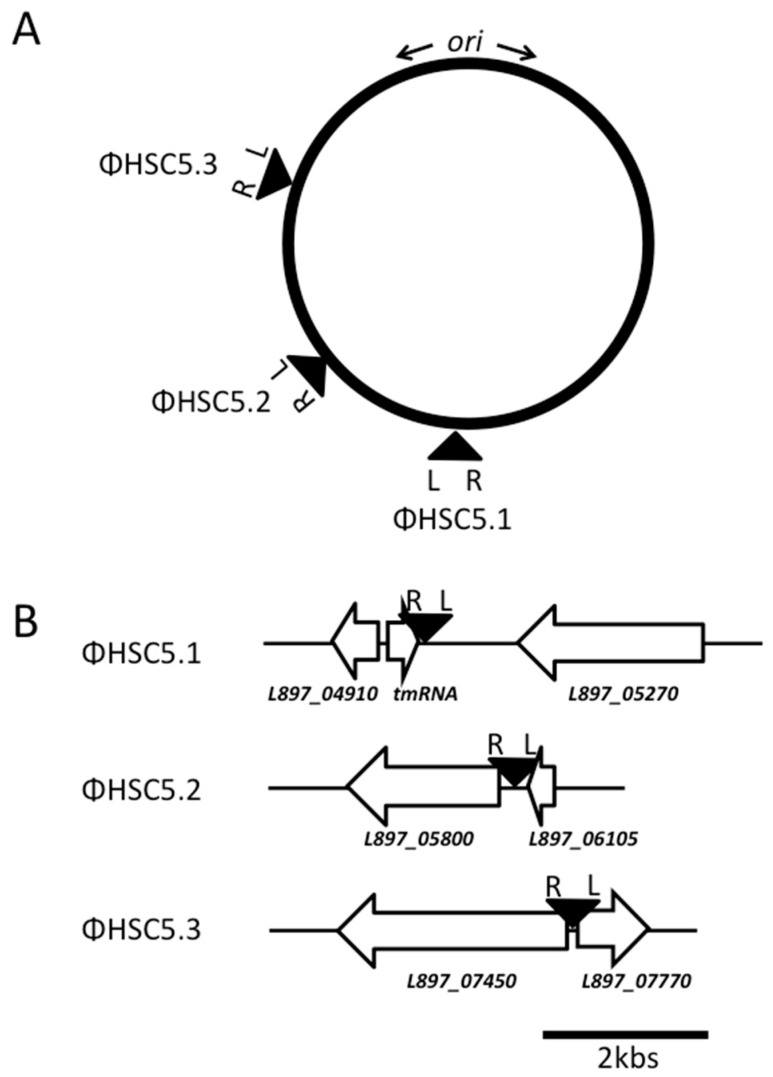
Prophage integration sites on the chromosome of *Streptococcus pyogenes* HSC5. (**A**) Positions of prophages on the *S. pyogenes* HSC5 genome. The circle represents the *S. pyogenes* chromosome. *ori* indicates the chromosomal replication origin for bidirectional DNA synthesis. The positions of the prophages, ΦHSC5.1, ΦHSC5.2, and ΦHSC5.3 on the chromosome are 31.8 min, 37.3 min, and 48 min, respectively. Inverted triangles indicate integration sites of the prophages. L denotes the position of *att*L next to integrase genes, and R denotes *att*R next to paratox genes; and (**B**) prophage integration sites in the HSC5 genome. White arrows indicate ORFs identified by locus tags (L897 numbers) of the *S. pyogenes* HSC5 genome.

The host strain, HSC5, has three prophages on its chromosome. We named them ΦHSC5.1, ΦHSC5.2, and ΦHSC5.3, based on the location on the chromosome (Figure 4). ΦHSC5.1 integrates immediately downstream of the tmRNA gene. In other strains, such as SF370, MGAS315, and NZ131, a gene of 621 nts encoding a hypothetical protein exists immediately downstream of the tmRNA gene in the same direction. However, the ORF does not exist in HSC5 because of a frame shift mutation in the middle of the gene. The flanking gene *L897_05270* encodes a putative maltodextrin phosphorylase. ΦHSC5.2 integrates at the IGR between *L897_05800* encoding a putative cation-transporting ATPase and *L897_06105* encoding a putative DNA binding protein. ΦHSC5.3 integrates at the IGR between the genes of putative excinuclease ABC subunit A (L897_07450, UvrA) and a putative divalent cation transport protein (L897_07770). The prophage deleted by in vitro passaging was ΦHSC5.3. The other two prophages, ΦHSC5.1 and ΦHSC5.2, were present on the chromosomes of all mutant strains (Figure 2 and Figure 3A). During lysogeny, a host gene could be disrupted by the insertion of a prophage and this disruption can cause the host’s phenotypic change. However, the phenotypic change of capsule production by ΦHSC5.3 is not due to a host gene disruption since the prophage ΦHSC5.3 is inserted between host genes. 

### 3.4. The Role of CvfA in Capsule Thermoregulation is Independent of the Curing of ΦHSC5.3

Previously, we searched for putative regulator genes whose disruption abolished capsule thermoregulation through transposon mutagenesis [10]. We screened mutants producing a large amount of capsule regardless of incubation temperature. Three chromosomal loci were identified from the transposon mutagenesis: capsule operon upstream IGR, the endoribonuclease gene *cvfA* (aka *rny*) and a prophage gene (*L897_07695*). We demonstrated that CvfA influences capsule thermoregulation at a post-transcriptional level [10].

Since ΦHSC5.3 can be spontaneously cured during cell handling, such as the process of transposon mutagenesis, we examined through PCR analysis if the transposon-generated mutants still carry ΦHSC5.3 (Figure 3B). The mutants with a transposon insertion in the *cvfA* gene and the prophage gene retained ΦHSC5.3. However, the mutant with a transposon insertion in the IGR immediately upstream of the capsule operon did not retain ΦHSC5.3. We also tested a previously created *cvfA*-directed disruption mutant [17] and this mutant also carried ΦHSC5.3. Taken together, these results indicate that the influence of CvfA on capsule thermoregulation is independent of the curing of ΦHSC5.3.

### 3.5. Disruption of the L897_07695 Gene in ΦHSC5.3 Confers the Same Capsule Thermoregulation-Negative Phenotype as That of the ΦHSC5.3-Cured Strains

The prophage gene identified from the transposon mutagenesis (*L897_07695*) is in ΦHSC5.3. In the transposon-generated mutant, a transposon was inserted between T78 and A79 in the *L897_07695* gene. The gene product is a putative DNA replication protein. To confirm the result of the transposon mutagenesis, we performed a directed insertional polar disruption of the gene through homologous recombination. Like the transposon insertion, the disruption by a plasmid insertion can cause the polarity of gene expression in a multi-gene operon. The resulting insertional disruption mutant also showed the same phenotype as that of the transposon-generated mutant (Figure 1). This result suggests that some genes in ΦHSC5 could be involved in capsule thermoregulation.

## 4. Discussion

Most *S. pyogenes* strains sequenced so far contain prophages in their chromosome [16]. The strain used in this study, HSC5, also harbors three prophages in its chromosome. Most streptococcal prophages carry virulence factors at one end of its genome. These virulence factors are DNases, pyrogenic exotoxins, such as superantigens and phospholipases, which are known to contribute to the pathogenic characteristic of *S. pyogenes* [18]. This study shows that streptococcal prophages can also control the expression of chromosome-encoded virulence factors. The prophage ΦHSC5.3 not only encodes DNase3 but also controls the expression of the capsule. The curing of ΦHSC5.3 abolishes capsule thermoregulation, so the mutant produces a large amount of capsules regardless of temperature, indicating that the presence of ΦHSC5.3 influences capsule production. The capsule seems not to be the only chromosomally-encoded virulence factor whose expression is controlled by a prophage. Previously, Spanier et al. reported that the expression of another major virulence factor, M protein, was also regulated by a prophage [19]. An M protein-negative strain can produce M protein by lysogenization with an appropriate bacteriophage. Additionally, curing of the bacteriophage reverted the M protein expression to the original status. They concluded that M protein expression is very low in the M protein-negative strain and lysogenization of the bacteriophage activates M protein expression by an unknown mechanism.

All three prophages in HSC5 have a similar genetic organization to the Siphoviridae family from low GC content Gram-positive bacteria [20]. These prophages have five major clusters in their genomes in the order of lysogeny, replication, packaging, structure, and lysis. The lysogeny cluster, the first cluster next to the *attL* site, starts with an integrase gene and contains genes for a characteristic *cI* family repressor and an antirepressor. The replication cluster follows the lysogeny cluster and contains DNA replication initiator proteins, recombination proteins, and/or methyltransferases. The packaging cluster includes a terminase, which is involved in site-specific binding and cutting of DNA. The structural cluster includes genes encoding the capsid, tail, and tail fibers of the phages. The lytic cluster is located just after the structural cluster and contains cell wall hydrolases (lysins), endopeptidases, and/or holins. The HSC5 prophages encode toxin genes in their genomes near the *attR* site (the opposite end of the integrase genes). ΦHSC5.1 has two putative streptococcal pyrogenic exotoxin genes consecutively (*L897_04920* and *L897_04925*). Interestingly, both ΦHSC5.2 and ΦHSC5.3 have the same toxin gene (*L897_07460 and L897_05810*) that encodes streptococcal extracellular DNase (mitogenic factor). The genes are 100% identical at the nucleotide level. The expression of these toxin genes and contribution to the virulence of HSC5 have not been studied yet. All of the prophages in HSC5 have a paratox gene next to the toxin genes. The paratox genes are highly-conserved open reading frames (ORFs) adjacent to the toxin genes in the majority of *S. pyogenes* prophages and suggested to be involved in the horizontal transfer of toxin genes between prophages through homologous recombination [21]. All HSC5 prophages are located in one half of the chromosome with the same orientation (Figure 4). The majority of their genes encoding structural proteins, lysins, and toxins are located to be transcribed in the direction of the chromosome replication, possibly to avoid collision between DNA and RNA polymerases [22,23]. The overall GC content of the three prophages is not different from that of the chromosome, which is 38.5%.

Many invasive strains have a mutation in the *covRS* genes [24], and produce an increased level of CovR-regulated virulence factors, including the capsule. The capsule-thermoregulated strains in our previous study are also invasive strains [10]. Thus, even though the capsule-thermoregulated strains do not produce a detectable level of the capsule at the host body temperature, capsule thermoregulation does not limit the strains to be invasive. After infection, if the capsule thermoregulation strains lose a prophage controlling capsule thermoregulation, and so produce the capsule at 37 °C, then the strains could survive better and become invasive.

Currently, what kind of prophages control capsule production are not known. None of the capsule-thermoregulated strains in our previous study have been sequenced, so how many and what types of prophages exist in the strains are not known. When we tested if a prophage exists at the same position where ΦHSC5.3 is inserted through PCR with the same primers that determine the presence of ΦHSC5.3 (Primer D set in Table 2), none of the strains had a prophage where ΦHSC5.3 is inserted. However, this cannot rule out that prophages similar to HSC5.3 inserting into other places regulate capsule thermoregulation in those strains.

Through transposon mutagenesis, we screened for the mutants overproducing the capsule regardless of incubation temperature to discover putative regulator genes for capsule thermoregulation [17]. The genes or DNA loci identified in the screen were *cvfA*, a prophage gene, and the IGR immediate upstream of the capsule genes. The prophage gene was *L897_07695* in ΦHSC5.3, and a transposon was inserted between T78 and A79 in the gene. The targeted disruption of *L897_07695* with a suicide vector also showed the same capsule thermoregulation phenotype as that of the transposon-generated mutant (Figure 1). The *L897_07695* gene is in the replication cluster in ΦHSC5.3, and forty-nine open reading frames exist downstream of the gene in the same direction, so a downstream gene, or genes, of *L897_07695* could be responsible for the mutant phenotype when the polarity of bacterial gene expression is considered. We are currently investigating to find the prophage gene, or genes, influencing capsule production. CvfA null mutants lose capsule thermoregulation and overproduce the capsule regardless of environmental temperature [10]. Since the prophage HSC5.3 can be spontaneously cured, we examined if the transposon-generated mutants of CvfA still carried the prophage. The prophage existed on the chromosome of the transposon-generated CvfA mutants (Figure 3B). This result indicates that the capsule thermoregulation-negative phenotype of the transposon-generated CvfA null mutants is not linked to spontaneous curing of ΦHSC5.3. This result was expected because add-back of the *cvfA* gene to the CvfA null strains reverted the phenotype of capsule thermoregulation [10]. Previously, the capsule operon upstream IGR was speculated to also be involved in capsule thermoregulation [10]. However, the prophage ΦHSC5.3 was not detected in the genome of the transposon mutant. Thus, the IGR may not be involved in capsule thermoregulation unless a transposon insertion in the IGR somehow triggers the curing of ΦHSC5.3.

Both CvfA and the prophage HSC5.3 are involved in capsule thermoregulation. A possible mechanism of capsule thermoregulation is that a regulatory factor is produced from a prophage gene whose transcript is processed by CvfA depending on environmental temperature. The regulatory molecule produced from the prophage would be a repressor because the capsule is produced at 37 °C after curing of the prophage ΦHSC5.3.

## Figures and Tables

**Table 1:** Bacterial strains.

Strain	Relevant Genotype/Description	Reference/Source
***Escherichia coli***		
TOP10	*mcrA* Δ(*mrr-hsdRMS-mcrBC*) φ80*lacZ*ΔM15 Δ*lacX*74 *nupG recA*1 *araD*139 Δ(ara-leu)7697 *galE*15 *galK*16 *rpsL*(Str^R^) *endA*1	Invitrogen
***Streptococcus pyogenes***		
HSC5	Wild-type, M14 serotype	[12]
CovRIFD	HSC5 strain with in-frame deleted *covR*. This strain has a deletion from the leucine codon (L39) to valine codon (V183) in the gene of CovR (228 amino acid long). This is the parental strain for most mutagenesis in this study.	[10]
ΩL897_07695	Strain of CovRIFD with the disruption of the prophage gene *L897_07695*	This study
CovRIFD:TnCvfA1 & CovRIFD:TnCvfA2	Strain of CovRIFD with a transposon insertion into *cvfA*	[10]
CovRIFD:TnL897_07695	Strain of CovRIFD with a transposon insertion into *L897_07695*	[10]
CovRIFD:TnHasAupIGR	Strain of CovRIFD with a transposon insertion into the intergenic region immediate upstream of *hasA*	[10]

**Table 2:** Primers used to determine the presence of prophages.

Name	Prophage	Forward	Reverse
A	ΦHSC5.1	5ΦHSC5.1 AGAAACAGGCGATGCCATAC	3ΦHSC5.1 CATTCATTTCATCCGACAGC
B	ΦHSC5.2	5ΦHSC5.2 GTCCACCGCTAAATCGAGAC	3ΦHSC5.2 ATTTCTCCACCGATTTCACG
C	ΦHSC5.3	5ΦHSC5.3 CAATTATGGGAGCGGCTATG	3ΦHSC5.3 ATCAAATTCTGTCGCCCAAG
D	ΦHSC5.3	5ΦHSC5.3del TTGCCCTTCCGCATAAATAG	3ΦHSC5.3del ATCAATGGTCCTCAAGGCAG

Primers are listed in the 5’ to 3’ direction.

## Supplementary Materials

The following are available online at http://www.mdpi.com/2073-4425/7/10/74/s1, Table S1: Mutation sites in the chromosome of spontaneous capsule thermoregulation mutants.

## Abbreviations

The following abbreviations are used in this manuscript:

CFU	Colony Forming Unit
IGR	Intergenic Region
IFD	In-Frame Deletion
OD	Optical Density
PBS	Phosphate-Buffered Saline
PCR	Polymerase Chain Reaction
WT	Wild Type
